# A mathematical model for varicella-zoster and HIV co-dynamic supported by numerical simulations

**DOI:** 10.1371/journal.pone.0299734

**Published:** 2024-03-01

**Authors:** Belela Samuel Kotola

**Affiliations:** Department of Mathematics, Oda Bultum University, Chiro, Ethiopia; Kaohsuing Medical University Hospital, TAIWAN

## Abstract

The prevalence of the varicella-zoster virus (VZV) and its correlation underscore its impact on a significant segment of the population. Notably contagious, VZV serves as a risk factor for the manifestation of HIV/AIDS, with its reactivation often signaling the onset of immunodeficiency. Recognizing the concurrent existence of these two diseases, this study focuses on the co-infection dynamics through a deterministic mathematical model. The population is categorized into seven exclusive groups, considering the complexities arising from the interplay of HIV and Zoster. We establish the non-negativity and boundedness of solutions, examine equilibrium points, calculate basic reproduction numbers via the next-generation matrix approach, and analyze the existence and local stabilities of equilibriums using the Routh-Hurwitz stability criteria. The numerical simulations reveal that the model converges to an endemic equilibrium point when the reproduction number exceeds unity. The primary objectives of this study are to comprehensively understand the transmission dynamics of HIV and Zoster in a co-infected population and to provide valuable insights for developing effective intervention strategies. The findings emphasize the importance of addressing these co-infections to mitigate their impact on public health.

## 1. Introduction

Microbes with the capacity to jeopardize health, including bacteria, fungi, viruses, and parasites, hold the capability to spread infectious diseases. Termed as commonplace ailments, these illnesses can be transmitted between hosts through diverse channels such as aerosol droplets, contaminated water or food, disease vectors, and via mother-to-child transmission [1–3].

The varicella-zoster virus (VZV) is accountable for causing both varicella (chickenpox) and shingles, also referred to as herpes zoster (known as Almaz bale Chira in Amharic). The occurrence of the varicella-zoster virus (VZV) is not directly associated with the birth rate in a population. Instead, it spreads through respiratory droplets and direct contact with an active herpes zoster infection [3].

The varicella-zoster virus is extremely easily transmitted and impacts illnesses that have a widespread impact on a significant portion of the population. Its occurrence usually aligns with the number of conceptions [2]. Nevertheless, individuals commonly develop lifelong immunity to VZV [3,4]. Additionally, the varicella-zoster virus (VZV), the initial indicator of the disease, can undergo reactivation due to infection with the human immunodeficiency virus (HIV) [5].

Impaired immune function can be apparent as a result of both sudden and prolonged lytic infections triggered by different viral agents, with a specific focus on herpes viruses. These alterations in the immune system may occur before the signs of AIDS become apparent. Notably, the varicella-zoster virus (VZV) is particularly noteworthy among these viral agents, as it can reactivate in individuals who are HIV-positive. Consequently, the simultaneous presence of HIV and Zoster is a plausible situation in a single person [3,6].

Co-infection is a combination of two heterogeneous strains or pathogens, either together or within a relatively brief amount of time before the first strain or pathogen establishes infection and develops an immune response. It is also the infection of a single host by two or more pathogen variants (strains) or two or more distinct pathogen species. Co-infection with multiple pathogen strains is especially common in pneumonia, but it can occur in a variety of other diseases as well [7–9]. Super-infection is described as a subsequent infection with a different strain and the ensuing immune response [9]. The concept of co-infection extends to the infection of a single host by multiple variants (strains) of a pathogen or even by different species of pathogens. This intricate interplay is particularly prevalent in conditions like pneumonia, and its occurrence is not limited to a specific set of diseases [8].

Additionally, the term "super-infection" refers to a subsequent infection caused by a different strain, indicating a separate stage in the host’s immune response. The successive introduction of diverse strains presents a distinct set of challenges and elicits responses within the host organism, thereby adding to the overall complexity of infectious diseases. [9].

This intricate dance between multiple pathogens and the host’s immune response underscores the dynamic nature of infectious diseases. Investigating these phenomena not only deepens our understanding of the intricacies of host-pathogen interactions but also holds potential implications for the development of targeted and more effective therapeutic interventions.

People with initial CD4 cell counts between 200 and 350, who show no detectable virus in their bloodstream a year after starting treatment, can expect a life span similar to that of the general population. To illustrate, men aged 35 or 50 might anticipate living until around 78 or 81 years, respectively. The term "durably undetectable" viral load refers to the absence of the virus in the blood for at least six months from the initial test without any detectable virus. Therefore, most individuals may need to adhere to treatment for a period ranging from 7 to 12 months to achieve and maintain a durably undetectable viral load [10].

Successful treatment has the capacity to significantly diminish the quantity of HIV in an individual’s bodily fluids to the extent that it becomes virtually impossible for them to transmit the virus to others. When the viral load is extremely low, it is deemed ’undetectable.’ By consistently adhering to a daily antiretroviral regimen, individuals with HIV can maintain an undetectable viral load, eliminating the risk of transmitting the virus to their non-HIV-infected sexual partners [11].

While many studies often concentrate on preventing or treating specific diseases, it is essential to tackle the increasing instances of individuals contending with multiple infections or diseases. Considerable research has been dedicated to comprehending the effects of various harmful microorganisms, such as viruses and bacteria, on both individuals and communities. As a result, there is an urgent requirement for a comprehensive model that clarifies how two different types of viruses can infect the same group of hosts [12–14]. Building upon the assumptions of this model, I have formulated the following framework to explore and address this complex interaction.

## 2. Model

### 2.1. Baseline and assumption for model formulation

- The human population distribution is homogeneous in each class
- those who have durably undetectable viral load do not transmit infection to others
- Individuals in each class are subject to a natural death rate.
- The population of human beings is variable.
- There is no dual-infection transmission simultaneously
- Vertical transmission has been considered for HIV.
- Since, HIV infection can lead to a weakened immune system, particularly a decline in CD4 T-cell counts, which are crucial for the body’s immune response. This weakened immune function can increase the risk of the reactivation of latent viruses, including the Varicella-Zoster Virus (VZV). As a result there is no permanent immunity for zoster infection.
- The vaccination wanes has been considered.

The study partitions the total human population at a given time t which is denoted by *N*(*t*), into seven exclusive classes depending on their infection status as follow.

**Susceptible class to both zoster and HIV** (S(t)): Individuals in this class are susceptible to both Zoster (Varicella-Zoster Virus) and HIV. They have not been previously infected with either virus, and they have not received the Zoster vaccine.

**Zoster vaccinated class (V(t)):** This class includes individuals who have been vaccinated against Zoster. Vaccination provides immunity to Zoster, reducing the risk of infection or, in some cases, modifying the course of the disease if breakthrough infection occurs.

**Zoster mono-infection class (Z(t)):** Individuals in this class are infected with Zoster but do not have an active HIV infection. They may have had VZV infection or developed reactivation of latent VZV.

**HIV mono-infected class (H(t)):** This class comprises individuals who are infected with HIV but do not have an active Zoster infection. They may be in various stages of HIV infection, and the class helps capture the dynamics of HIV transmission.

**HIV and Zoster co-infection class (C(t)):** Individuals in this class are co-infected with both HIV and Zoster.

**Class of those with durably undetectable viral load (D(t)):** This class represents individuals with HIV who have achieved durably undetectable viral loads through successful antiretroviral therapy (ART). These individuals are effectively controlling their HIV infection, reducing the risk of transmission to others.

**Recovered class (R(t)):** Individuals in this class have recovered from Zoster infection.


N(t)=S(t)+Z(t)+H(t)+C(t)+D(t)+V(t)+R(t). (1)

Since both zoster and HIV are chronic diseases the susceptible individuals acquire them at the standard incidence rate given as follows respectively.


$$\lambda_{h}(t)=\frac{\beta_{1}}{N}(H(t)+D(t)+\omega_{1}C(t)).$$ (2)

$$\lambda_{z}(t)=\frac{\beta_{2}}{N}(Z(t)+\omega_{2}C(t)).$$ (3)

Where ω_1_, ω_2_≥1 are the modification parameter that represents the infectiousness degree.

β_1_ and β_2_ are the transmission rate of HIV and zoster respectively. In assessing the proposed deterministic model for HIV and zoster co-infection dynamics, key benchmarks include validating equilibrium points against established epidemiological models, ensuring a mathematically and epidemiologically well-posed region, and confirming the reproduction number’s significance in line with known theoretical values [15].

**Fig 1:**
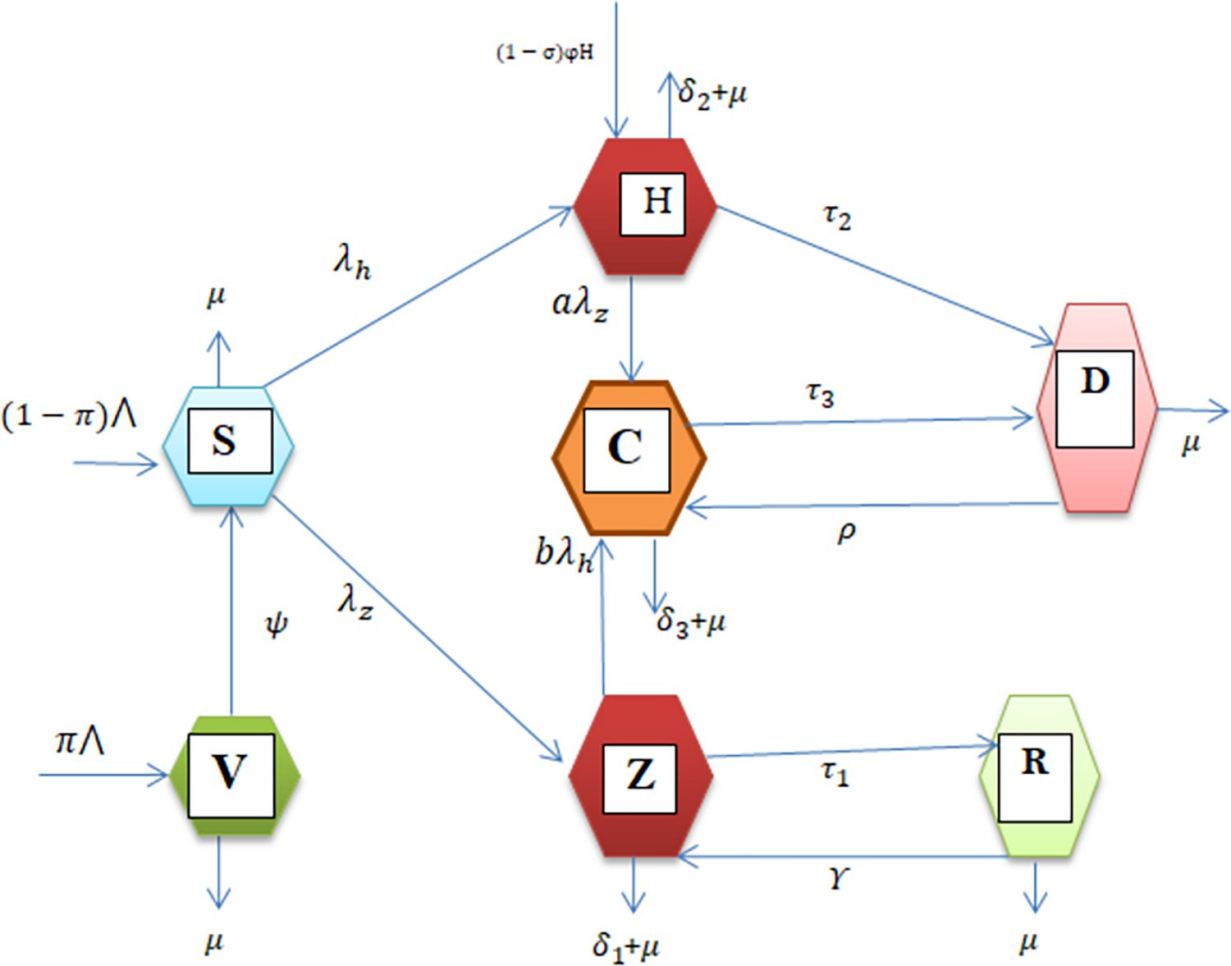
Flow chart.

Using parameters and variables in Table 1, the model schematic diagram for the transmission dynamics of Zoster and HIV super-infection is given in Fig 1.

**Table 1:** Biological representation of models’ parameters and variables.

*Parameter Biological definitions*		*Unit*
*μ*	Human natural mortality rate	Time^−1^
⋀	Human recruitment rate	Size*Time^−1^
*π*	Portion of vaccination of Zoster	Time^−1^
*ψ*	Vaccination wanes rate	Time^−1^
*a*	Modification	Time^−1^
*b*, c	Modification	Time^−1^
*δ*_1_	zoster death rate	Time^−1^
*δ*_2_	HIV death rate for Unaware	Time^−1^
*δ*_3_	zoster and HIV co-infection death rate	Time^−1^
*γ*	The rate at which Zoster recovered individual re-infected	Time^−1^
*ρ*	The rate at which HIV infected with undetectable viral load become re-infected by zoster	Time^−1^
*β*_1_	HIV Transmission rate	Size*Time^−1^
*β*_2_	Zoster Transmission rate	Size*Time^−1^
*τ*_1_	Zoster treatment rate	Time^−1^
*τ*_2_	HIV treatment rate	Time^−1^
*τ*_3_	The rate at which Zoster and HIV co-infected individuals treated	Time^−1^
*σ*	Probability of death of newborns infected with HIV at birth	Time^−1^
*φ*	Rate of Mother to Child Transmission rate of HIV	Time^−1^
*S*	Susceptible class	Time^−1^
*H*	HIV infectious Class	Time^−1^
*Z*	Zoster infectious Class	Time^−1^
*C*	Zoster and HIV Co- co-infected class	Time^−1^
*D*	Class of those who are undetectable viral load	Time^−1^
*V*	Class of those who take vaccination of Zoster	Time^−1^
*R*	A group of people recovered from Zoster disease	Time^−1^

The above flow chart is managed by the following dynamical system.


$$\begin{array}{l} \;\frac{dS}{dt}=(1-\pi )⋀+\psi V–(\lambda_{h}+\lambda_{z}+\mu )S,\\ \frac{dV}{dt}=\pi ⋀-(\psi +c\lambda_{h}+\mu )V,\\ \frac{dH}{dt}=\lambda_{h}S+c\lambda_{h}V+(1-\sigma )\varphi H-(a\lambda_{z}+\tau_{2}+\delta_{2}+\mu )H,\\ \;\frac{dZ}{dt}=\lambda_{z}S+\Upsilon R-(b\lambda_{h}+\tau_{1}+\delta_{1}+\mu )Z,\\ \;\frac{dC}{dt}=a\lambda_{z}H+b\lambda_{h}Z+\rho D–(\tau_{3}+\delta_{3}+\mu )C,\\ \;\frac{dD}{dt}=\tau_{2}H+\tau_{3}C-(\rho +\mu )D,\\ \;\frac{dR}{dt}=\tau_{1}Z-(\Upsilon +\mu )R. \end{array}\}$$ (4)

The above dynamical system of equations captures the interactions between susceptible, infected, co-infected, treated, and recovered individuals for both Varicella-Zoster and HIV. The parameters in the equations govern the rates of transmission, recovery, progression, treatment initiation, and natural death, providing a comprehensive representation of the epidemiological dynamics of these two infections within a population. The model allows for the exploration of different scenarios and interventions to understand their impact on the dynamics of Varicella-Zoster and HIV co-infections.

## 3. Model analysis

In this subsection, I have looked at the model’s qualitative behavior and presented the analysis of the proposed model.

### 3.1 Well-posedness of the model

**Theorem 1:** The solutions of the system of Eq (4) are positive, unique, and bounded in the region

$$\Omega =\left\{(S,V,H,Z,C,D,R)\epsilon R_{+}^{7}:\;0\leq N\leq \frac{⋀}{\mu }\right\}.$$ (5)

**Proof:** The Picard–Lindelöf theorem [16], which is a fundamental result in the theory of ordinary differential equations guarantees the existence and uniqueness of solutions to certain initial value problems for ordinary differential equations when the functions involved are locally Lipschitz continuous. In this case, the functions on the right-hand side of Eq (4) are are C^1^ on $R_{+}^{7}$ (continuously differentiable), satisfying the conditions required by the Picard–Lindelöf theorem. Therefore, a unique solution to the system of equations exists.

**Non-negativity of Functions:** Proposition A.1 in reference [17] is mentioned to assert that the functions f_i_(S,V,H,Z,C,D,R) (the right-hand sides of the differential equations) are non-negative whenever x∈[0,∞]^7^ and x_i_ = 0. This property is crucial in the context of the model. It means that, in regions where any of the components S,V,H,Z,C,D,R are zero, the corresponding rates of change are non-negative. This is often a desired property, especially in epidemiological models, where negative values for variables like population or compartments are not physically meaningful.

**Boundedness of Solutions:** The proof then moves on to demonstrate that the solutions to the system of equations are bounded. It is stated that the functions f_i_(S,V,H,Z,C,D,R) have the property

$$f_{i}(S,V,H,Z,C,D,R)\geq 0.$$ (6)

(as per proposition A.1). This non-negativity property, combined with the Picard–Lindelöf theorem, ensures that the solutions remain in the region [0,∞]^7^ for all t≥0. The specific bound for the total population N is given in Eq (7), where

$$\frac{dN}{dt}\leq ⋀-\mu N.$$ (7)

The solution for N(t) is then provided as $N(t)\leq {N_{0}e}^{-\mu t}+\frac{⋀}{\mu }(1-e^{-\mu t}).$ This solution implies that the population N(t) is bounded within the interval [0,Λ/μ]. Therefore, the solution of the system of equations in (4) exists, unique, and bounded in a feasible region *Ω*.

Biologically, the positivity of solutions ensures that populations such as susceptible, infected, and co-infected individuals remain non-negative, aligning with the realistic nature of epidemiological scenarios. Uniqueness indicates a well-defined trajectory for each population, enhancing predictability, while bounded solutions signify that the modeled populations do not exhibit uncontrolled growth, providing stability and aligning with real-world limitations.

### 3.2 Disease-free equilibrium point

The disease-free equilibrium point (*E*^0^) of the system is obtained by making all the equations equal to zero, by assuming that there is no disease in communities (i.e. H = Z = C = 0).

Thus, the disease-free equilibrium point of the system in Eq (4) is given by:

$$E^{0}=(S^{0},V^{0},H^{0},Z^{0},C^{0},D^{0},R^{0})=(\frac{(\psi +\mu )(1-\pi )⋀+\pi ⋀\psi }{(\psi +\mu )\mu },\frac{\pi ⋀}{\psi +\mu },0,0,0,0,0).$$ (8)

### 3.3 Reproduction numbers

Reproduction number (ℛ_0_), is the estimated number of secondary cases that a normal infected person will cause in a completely susceptible society [15,16]. To determine the reproduction number, it is essential to isolate recently infectious changes from all other changes in the host population. Let ${V^{+}}_{i}(x):$ represent the rate of people moving into compartment i, ${V^{-1}}_{i}(x):$ represent the rate of people moving out of compartment i, and ℱ_*i*_(*x*): represent the rate at which new infectious diseases arise in compartment i.

Then $V_{i}(x)={V^{-}}_{i}(x)-{V^{+}}_{i},F=\left[\frac{\partial F_{i}}{\partial X_{j}}(X_{o})\right]$ and $V=\left[\frac{\partial V_{i}}{\partial X_{j}}(X_{o})\right]$, where *F* and *V* are *mxm* matrix with *m* is number of infected compartment. Then *Fv*^−1^ is the next generation matrix and the spectral radius of next generation matrix is the needed reproduction number [13,15]. Consequently, we do have the following from our system. For this study, we have analyzed the three possible reproduction numbers as follows, since we are examining two diseases in a single model.

#### 3.3.1 HIV reproduction number (ℛ_*h*_)

From equation of system(4) we do have the HIV sole infected model of the following categories.


$${F_{i}}^{\left(x\right)}=\left[\begin{array}{c} \lambda_{h}S\\ 0 \end{array}\right]\;\mathrm{and}\;V_{i}(x)=[\begin{array}{c} (\tau_{2}+\delta_{2}+\mu )H-(1-\sigma )\varphi H\\ (\rho +\mu )D-\tau_{2}H \end{array}].$$ (9)

Since reproduction number is manipulated at disease free equilibrium point, *N* = *S* and the infected compartments are H = D = 0. … … … … …(10)

$$Thus\;F=\left[\begin{array}{cc} \beta_{1} & \beta_{1}\\ 0 & 0 \end{array}\right]\;\mathrm{and}\;V=[\begin{array}{cc} \tau_{2}+\delta_{2}+\mu -(1-\sigma )\varphi & 0\\ -\tau_{2} & \rho +\mu \end{array}],$$ (10)

$$V^{-1}=(\begin{array}{cc} \frac{1}{\tau_{2}+\delta_{2}+\mu -(1-\sigma )\varphi } & 0\\ \frac{\tau_{2}}{(\tau_{2}+\delta_{2}+\mu )(\rho +\mu )-(\rho +\mu )(1-\sigma )\varphi } & \frac{1}{\rho +\mu } \end{array}).$$

$$Then\;{FV}^{-1}=(\begin{array}{cc} (\frac{\beta_{1}}{\rho +\mu })\frac{\left(\rho +\mu +\tau_{2}\right)}{(\tau_{2}+\delta_{2}+\mu )-(1-\sigma )\varphi } & \frac{\beta_{1}}{\rho +\mu }\\ O & 0 \end{array}).$$ (11)

Therefore, the reproduction number of HIV sole infected sub model is

$$R_{h}=\left(\frac{\beta_{1}}{\rho +\mu }\right)\frac{\left(\rho +\mu +\tau_{2}\right)}{(\tau_{2}+\delta_{2}+\mu )-(1-\sigma )\varphi }.$$ (12)

#### 3.3.2 Zoster reproduction number (ℛ_*z*_)

From equation of system (4) we do have the zoster sole infected model with the following categories.


$$F_{i}(x)=[\lambda_{z}S]\;and\;V_{i}(x)=(\tau_{1}+\delta_{1}+\mu )Z.$$ (13)

Since reproduction number is manipulated at disease free equilibrium point, *N* = *S*+*V* and the infected compartment is Z = 0

$$Thus\;F=\frac{\beta_{2}(\psi +\mu )\mu }{(\psi +\mu )(1-\pi )⋀+\pi ⋀\psi +\pi ⋀\mu }\;\mathrm{and}\;V=(\tau_{1}+\delta_{1}+\mu ),$$ (14)

$V^{-1}=\frac{1}{\tau_{1}+\delta_{1}+\mu }$.


$$Then\;{FV}^{-1}=\frac{\beta_{2}(\psi +\mu )\mu }{\left[(\psi +\mu )(1-\pi )⋀+\pi ⋀\psi +\pi ⋀\mu ](\tau_{1}+\delta_{1}+\mu \right)}.$$ (15)

Therefore, the reproduction number of zoster sole infected sub model is

$$R_{z}=\frac{\beta_{2}(\psi +\mu )\mu }{\left[(\psi +\mu )(1-\pi )⋀+\pi ⋀\psi +\pi ⋀\mu ](\tau_{1}+\delta_{1}+\mu \right)}$$ (16)

#### 3.3.3 Reproduction number of full model (ℛ_*hz*_)

Using the procedures in above Sub subsection of (3.3.1) and (3.3.2) and

$$F_{i}(x)=\left[\begin{array}{c} \lambda_{h}S\\ \lambda_{z}S\\ 0\\ 0 \end{array}\right]\;\mathrm{and}\;V_{i}(x)=[\begin{array}{c} (a\lambda_{z}+\tau_{2}+\delta_{2}+\mu )H-(1-\sigma )\varphi H\\ (b\lambda_{h}+\tau_{1}+\delta_{1}+\mu )Z-\Upsilon R\\ (\tau_{3}+\delta_{3}+\mu )C-a\lambda_{z}H-b\lambda_{h}Z-\rho D\\ (\rho +\mu )D-\tau_{2}H-\tau_{3}C \end{array}].$$ (17)

Since reproduction number is manipulated at disease free equilibrium point, *N* = *S*+*V* and the infected compartments are *H* = Z = C = D = 0.


$$Thus\;F=\left[\begin{array}{cccc} \frac{\beta_{1}S}{N} & 0 & \frac{\beta_{1}S}{N} & \frac{\beta_{1}S}{N}\\ 0 & \frac{\beta_{2}S}{N} & \frac{\beta_{2}S}{N} & 0\\ 0 & 0 & 0 & 0\\ 0 & 0 & 0 & 0 \end{array}\right]\;and$$

$$V=\left[\begin{array}{cccc} (\tau_{2}+\delta_{2}+\mu )-(1-\sigma )\varphi & 0 & 0 & 0\\ 0 & \left(\tau_{1}+\delta_{1}+\mu \right) & 0 & 0\\ 0 & 0 & \left(\tau_{3}+\delta_{3}+\mu \right) & -\rho \\ -\tau_{2} & 0 & -\tau_{3} & \left(\rho +\mu \right) \end{array}\right]$$ (18)

$$V^{-1}=(\begin{array}{cccc} \frac{1}{\mu -(1-\sigma )\varphi +\delta_{2}+\tau_{2}} & 0 & 0 & 0\\ 0 & \frac{1}{\mu +\delta_{1}+\tau_{1}} & 0 & 0\\ \frac{\rho \tau_{2}}{\left(\mu -(1-\sigma )\varphi +\delta_{2}+\tau_{2})(\mu^{2}+\mu \rho +\mu \delta_{3}+\rho \delta_{3}+\mu \tau_{3}\right)} & 0 & \frac{\mu +\rho }{\mu^{2}+\mu \rho +\mu \delta_{3}+\rho \delta_{3}+\mu \tau_{3}} & \frac{\rho }{\mu^{2}+\mu \rho +\mu \delta_{3}+\rho \delta_{3}+\mu \tau_{3}}\\ \frac{\tau_{2}(\mu +\delta_{3}+\tau_{3})}{\left(\mu -(1-\sigma )\varphi +\delta_{2}+\tau_{2})(\mu^{2}+\mu \rho +\mu \delta_{3}+\rho \delta_{3}+\mu \tau_{3}\right)} & 0 & \frac{\tau_{3}}{\mu^{2}+\mu \rho +\mu \delta_{3}+\rho \delta_{3}+\mu \tau_{3}} & \frac{\mu +\delta_{3}+\tau_{3}}{\mu^{2}+\mu \rho +\mu \delta_{3}+\rho \delta_{3}+\mu \tau_{3}} \end{array}).$$

$$FV^{-1}=(\begin{array}{cccc} \frac{a}{\mu -(1-\sigma )\varphi +\delta_{2}+\tau_{2}} & 0 & 0 & 0\\ 0 & \frac{b}{\mu +\delta_{1}+\tau_{1}} & 0 & 0\\ 0 & 0 & 0 & 0\\ 0 & 0 & 0 & 0 \end{array}).$$ (19)

The corresponding Eigenvalues is given by $\left\{0,0,\frac{\frac{\beta_{1}S}{N}}{\mu -\varphi +\sigma \varphi +\delta_{2}+\tau_{2}},\frac{\frac{\beta_{2}S}{N}}{\mu +\delta_{1}+\tau_{1}}\right\}$.

Thus the reproduction number of the full model is

$$R_{hz}=Max\{\frac{(\mu (1-\pi )+\psi )\beta_{1}}{\left(\mu +\psi )(\mu -\varphi +\sigma \varphi +\delta_{2}+\tau_{2}\right)},\frac{(\mu (1-\pi )+\psi )\beta_{2}}{\left(\mu +\psi )(\mu +\delta_{1}+\tau_{1}\right)}\}.$$ (20)

### 3.4 Equilibrium points and their stabilities

#### 3.4.1 Disease-free equilibrium point and its stability

3.4.1.1 Local stability of disease-free equilibrium point

**Theorem 2:** The disease-free equilibrium point ***E***^0^ of the model in system (4) is locally asymptotically stable if the effective reproduction number **ℛ**_***hz***_<1 and is unstable if **ℛ**_***hz***_>1.

Proof:

The Jacobian matrix *J*(*E*^0^) of the model concerning (*S*,*V*,*H*,*Z*,*C*,*D*,*R*) at the disease-free equilibrium point is the following.


$$J(E^{0})=\left[\begin{array}{ccccccc} –\mu & \psi & –A & –BS & –A\omega_{1}–B\omega_{2} & –A & 0\\ 0 & -C & 0 & 0 & 0 & 0 & 0\\ 0 & 0 & D & 0 & 0 & 0 & 0\\ 0 & 0 & 0 & -E & 0 & 0 & \Upsilon \\ 0 & 0 & 0 & 0 & –F & \rho & 0\\ 0 & 0 & \tau_{2} & 0 & \tau_{3} & -G & 0\\ 0 & 0 & 0 & \tau_{1} & 0 & 0 & -H \end{array}\right]$$ (21)

Where, A = $\frac{\beta_{1}}{N}S$, B = $\frac{\beta_{2}}{N}S$, C = (*ψ*+*μ*), D = (1−*σ*)*φ*−(*τ*_2_+*δ*_2_+μ), E = (*τ*_1_+*δ*_1_+μ),

F = (*τ*_3_+*δ*_3_+*μ*), G = (*ρ*+*μ*), H = (*γ*+*μ*).

Then Characteristic equation at DFE is given by

$$(-C-\lambda )(D-\lambda )(-\lambda +\mu –)\left((-E-\lambda )(-H-\lambda )(Gx+\lambda^{2}-FG+F\lambda +\rho \tau_{3})(1-{\Upsilon \tau }_{1})\right)=0$$

Then the solution of corresponding characteristic equation is

λ=D,λ=−(μ+ψ),λ=−μ,λ=−E,λ=(μ+Υ).

$$\mathrm{or}\;\lambda^{2}+(\mu +\rho -(\tau_{3}+\delta_{3}+\mu ))\lambda +(\tau_{3}+\delta_{3}+\mu )(\mu +\rho )+\rho \tau_{3}=0.$$ (22)

Here, negative Eienvalues has been obtained except for the expression of in Eq (22).

Upon applying the Routh Hurwitz stability requirements to Eq (22) it was found that there is no sign change in the first column of the Routh Hurwitz array for **ℛ**_***hz***_<1.

Therefore, **ℛ**_***hz***_<1, the dynamical system’s DFE equilibrium point is locally asymptotically stable[11,13]. As can be shown from the numerical simulation Section, the model’s disease-free equilibriums are globally asymptotically stable whenever the associated effective reproduction numbers values are less than unity.

Biologically, if the effective reproduction number (**ℛ**_**hz**_) is less than 1, the disease-free equilibrium point is locally stable, indicating that the diseases are under control, and outbreaks are unlikely. Conversely, if **ℛ**_**hz**_ exceeds 1, the equilibrium is unstable, suggesting the potential for sustained transmission and outbreaks. This information is crucial for public health efforts, providing a threshold for intervention effectiveness and serving as an early warning system for potential disease outbreaks within the population.

#### 3.4.2 Endemic equilibrium point and its stability

The endemic equilibrium point of the dynamical system of (**4**) is obtained by making right side of the system equal to zero providing that **H≠0, Z≠0, D≠0** and **C≠0**.

We have supposed the endemic equilibrium point of the model is denoted by ***E***^**0**^ = (S*,V*,H*,Z*,C*,D*,R*) and the corresponding forces of infection are:

$${\lambda_{h}}^{*}(t)=\frac{\beta_{1}}{N}(H^{*}(t)+D^{*}(t)+\omega_{1}C^{*}(t))\;and\;{\lambda_{Z}}^{*}(t)=\frac{\beta_{2}}{N}(Z^{*}(t)+\omega_{2}C^{*}(t)).$$

$$S^{*}=\frac{(1-\pi )⋀}{\left({\lambda_{h}}^{*}+{\lambda_{Z}}^{*}+\mu \right)}+\frac{\left(\psi \right)}{\left({\lambda_{h}}^{*}+{\lambda_{Z}}^{*}+\mu \right)}(\frac{\pi ⋀}{\left(\psi +\mu \right)}),\;V^{*}=\frac{\pi ⋀}{\left(\psi +\mu \right)},$$

$$H^{*}=\left(\frac{1}{(a{\lambda_{Z}}^{*}+\tau_{2}+\delta_{2}+\mu )-(1-\sigma )\varphi }\right)\left(\frac{(1-\pi )⋀\lambda_{h}}{\left({\lambda_{h}}^{*}+{\lambda_{Z}}^{*}+\mu \right)}+\frac{\psi \lambda_{h}}{\left(\lambda_{h}+{\lambda_{Z}}^{*}+\mu \right)}(\frac{\lambda_{h}\pi ⋀}{\left(\psi +\mu \right)})\right),Z^{*}=(\frac{(\pi -1)⋀{\lambda_{Z}}^{*}}{\left(\lambda_{h}+\lambda_{z}+\mu \right)}-\frac{\psi {\lambda_{Z}}^{*}}{(\lambda_{h}+\lambda_{z}+\mu )}(\frac{\pi ⋀}{\left(\psi +\mu \right)}))(\frac{\left(\Upsilon +\mu \right)}{\Upsilon \tau_{1}-(\Upsilon +\mu )(b{\lambda_{h}}^{*}+\tau_{1}+\delta_{1}+\mu )}),$$

$$D^{*}=\frac{\tau_{2}H^{*}+\tau_{3}C^{*}}{\left(\rho +\mu \right)},C^{*}=\frac{a\lambda_{z}H^{*}+b\lambda_{h}Z^{*}+\rho D^{*}}{\left(\tau_{3}+\delta_{3}+\mu \right)},$$

$$R^{*}=\left(\frac{\tau_{1}}{\left(\Upsilon +\mu \right)}\right)\left(\frac{(\pi -1)⋀{\lambda_{Z}}^{*}}{\left(\lambda_{h}+\lambda_{z}+\mu \right)}-\frac{\psi {\lambda_{Z}}^{*}}{\left(\lambda_{h}+\lambda_{z}+\mu \right)}(\frac{\pi ⋀}{\left(\psi +\mu \right)})\right)(\frac{\left(\Upsilon +\mu \right)}{\Upsilon \tau_{1}-(\Upsilon +\mu )(b{\lambda_{h}}^{*}+\tau_{1}+\delta_{1}+\mu )}).$$

Biologically, the endemic equilibrium reflects the dynamic equilibrium reached between the forces driving the transmission of the diseases (such as infection rates, recovery rates, and population structure) and those limiting their spread (such as immunity, interventions, and natural death). Understanding the properties and stability of the endemic equilibrium is crucial for assessing the long-term impact of infectious diseases on a population and for guiding public health strategies to control or manage these diseases.

### 3.5 Sensitivity analysis

Here, I’ve demonstrated how important each parameter is to the spread of the disease using the process of sensitivity indices of the effective reproduction number of the zoster and HIV co-infection model to all pertinent parameters. When compared to all other parameters, the most sensitive parameter’s sensitivity index has a larger magnitude. The sensitivity studies indicated below have been carried out for ℛ_1_ and ℛ_2_.

1. $SI(\psi )=(\frac{\partial R_{1}}{\partial \psi })*(\frac{\psi }{R_{1}}).⟹SI(\psi )=\frac{\psi (\mu -\mu (1-\pi ))}{\left(\mu +\psi )(\psi +\mu (1-\pi )\right)}$.
2. $SI(\pi )=(\frac{\partial R_{1}}{\partial ⋀})*(\frac{⋀}{R_{1}}).⟹SI(⋀)=1$.
3. $SI(\varphi )=(\frac{\partial R_{1}}{\partial \varphi })*(\frac{\varphi }{R_{1}}).⟹SI(\varphi )=-\frac{(\sigma -1)\varphi }{\mu -\varphi +\sigma \varphi +\delta_{2}+\tau_{2}}$.
4. $SI(\beta_{1})=(\frac{\partial R_{1}}{\partial \beta_{1}})*(\frac{\beta_{1}}{R_{1}}).⟹SI(\beta_{1})=\frac{\psi +\mu [1-\pi ]}{\left(\mu +\psi )(\mu -\varphi +\sigma \varphi +\delta_{2}+\tau_{2}\right)}(\frac{\beta_{1}}{R_{1}})=1$.
5. $SI(\sigma )=(\frac{\partial R_{1}}{\partial \sigma })*(\frac{\sigma }{R_{1}})$.
  $SI(\sigma )=-\frac{\varphi \beta_{1}(\psi +\mu [1-\pi ])}{(\mu +\psi ){\left(\mu -\varphi +\sigma \varphi +\delta_{2}+\tau_{2}\right)}^{2}}(\frac{\sigma }{R_{1}}).⟹SI(\sigma )=-\frac{\sigma \varphi }{\mu -\varphi +\sigma \varphi +\delta_{2}+\tau_{2}}$.
6. $SI(\beta_{2})=(\frac{\partial R_{2}}{\partial \sigma })*(\frac{\beta_{2}}{R_{2}})⟹SI(\beta_{2})=\frac{\psi +\mu [1-\pi ]}{\left(\mu +\psi )(\mu +\delta_{1}+\tau_{1}\right)}(\frac{\beta_{2}}{R_{2}})=1$.

**Fig 2:**
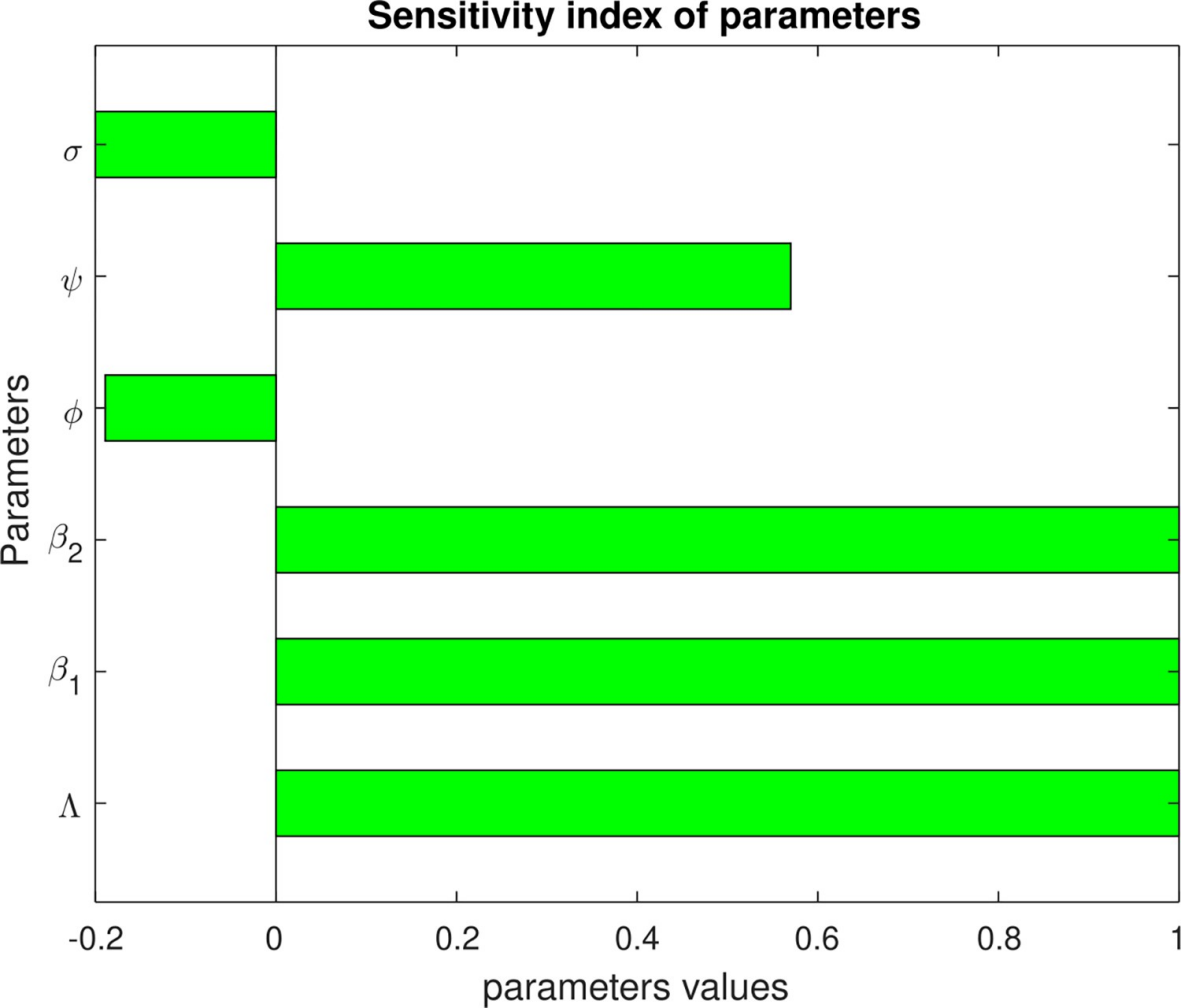
Sensitivity analysis plot.

In biological points of views, sensitivity analysis is a valuable tool in scientific modeling and decision-making, offering insights into the factors that significantly influence outcomes and enhancing the reliability and robustness of models. Using the following parameters values, I have described the influence of each parameters using the below Fig 2.

The above Fig 2 is obtained in consideration of ℛ>1 mean that, when the diseases exist or prevail in the population. In this study, we used the parameter values in Table 2 above and obtained the indices in Fig 2. The recruitment rate, transmission rate and portion of vaccination have the greatest impact on the basic reproduction number of zoster and HIV.

**Table 2:** Parameter values used for the model.

Parameters	Description	values	Unit	Sources
*μ*	Human natural mortality rate	0.1	Time^−1^	[14]
⋀	Human recruitment rate	100	Size*Time^−1^	[16,17]
*π*	Portion of vaccination of Zoster	0.8	Time^−1^	Estimated from [18,19]
*ψ*	Vaccination wanes rate	0.35	Time^−1^	Estimated
*a*	Modification	1	Time^−1^	Estimated
*b*	Modification	1	Time^−1^	Estimated
*δ*_1_	zoster death rate	0.32	Time^−1^	Estimated from [17]
*δ*_2_	HIV death rate for Unaware		Time^−1^	
*δ*_3_	zoster and HIV co-infection death rate	0.3	Time^−1^	Estimated from [14,17]
*γ*	The rate at which Zoster recovered individual re-infected	0.65	Time^−1^	Estimated from [17]
*ρ*	The rate at which HIV infected with undetectable viral load become re-infected by zoster	0.32	Time^−1^	Estimated
*β*_1_	HIV Transmission rate	2.5	Size*Time^−1^	Estimated from [18]
*β*_2_	Zoster Transmission rate	3.5	Size*Time^−1^	[17]
*τ*_1_	Zoster treatment rate	0.87	Time^−1^	Estimated from [15]
*τ*_2_	HIV treatment rate	0.45	Time^−1^	Estimated from [20]
*τ*_3_	The rate at which Zoster and HIV co-infected individuals treated	0.53	Time^−1^	Estimated from [19]
*σ*	Probability of death of newborns infected with HIV at birth	0.35	Time^−1^	Estimated
*φ*	Rate of Mother to Child Transmission rate of HIV	0.6	Time^−1^	Estimated

### 3.6 Numerical simulation

Some differential equations are challenging to solve analytically. In this scenario, a numerical simulation of the system is required. Moreover, numerical simulations are a crucial step in validating a mathematical model’s predictions regarding co-infection dynamics. The process involves solving the system of differential equations numerically, simulating the behavior of the model over time, and comparing the simulation results to relevant empirical data or expectations [10]. Even if there are many methods for solving system of non linear equation for investigation of parameter effect like [18,19], we employed numerical simulation to examine the effects of a variety of characteristics (parameters) associated with the Zoster and HIV co-infection sickness, such as treatment rate, treatment rate, vaccination effect, and vaccination wane rate.

**Fig 3:**
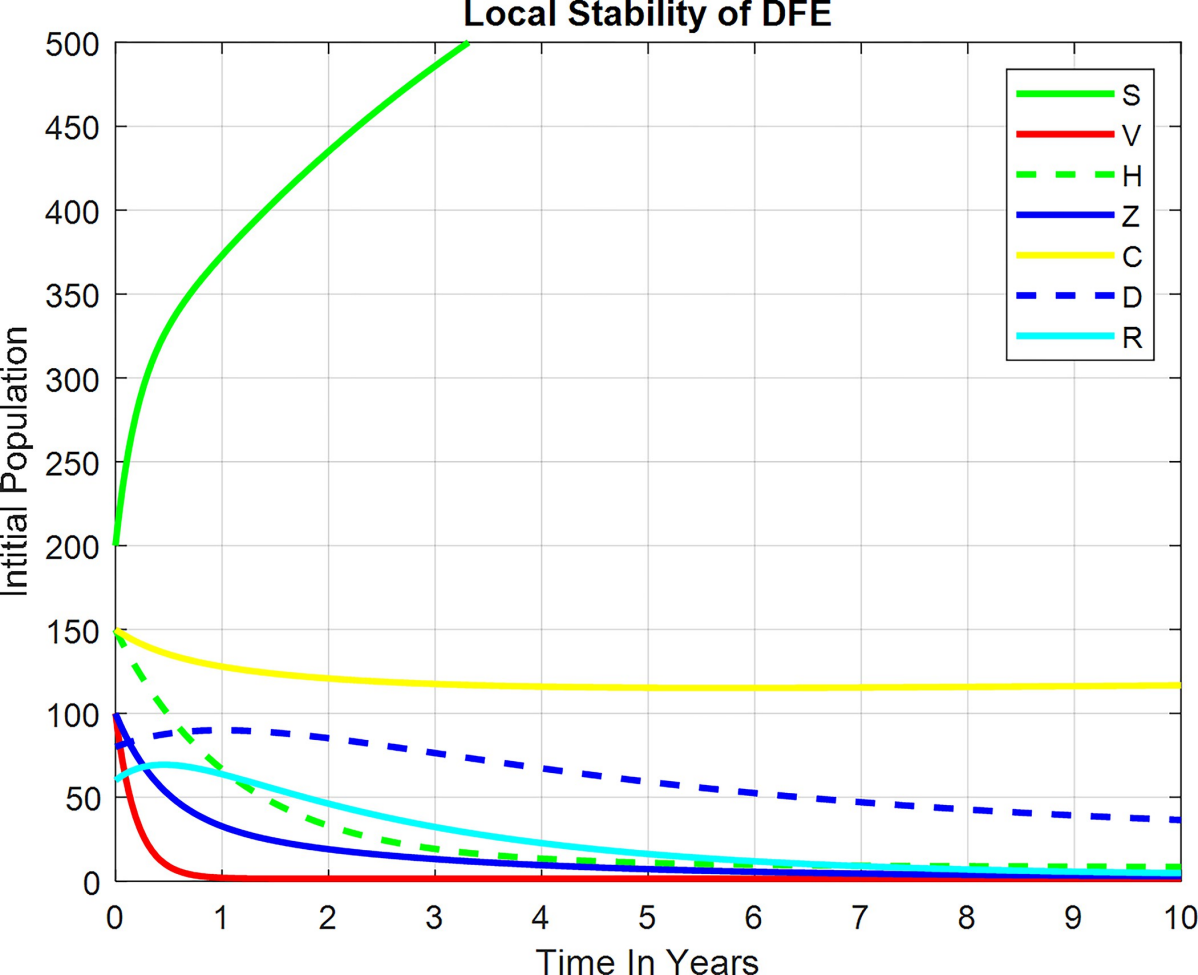
Local stability of DFE.

As we can see from the plot in Fig 3, the DFE point of the system is locally asymptotically stable for less than unit reproduction. Moreover, it depicts the biological meaning of the expansion and spread of Zoster and HIV co-infections, which occur regularly throughout a population that is confined to a specific location.

**Fig 4:**
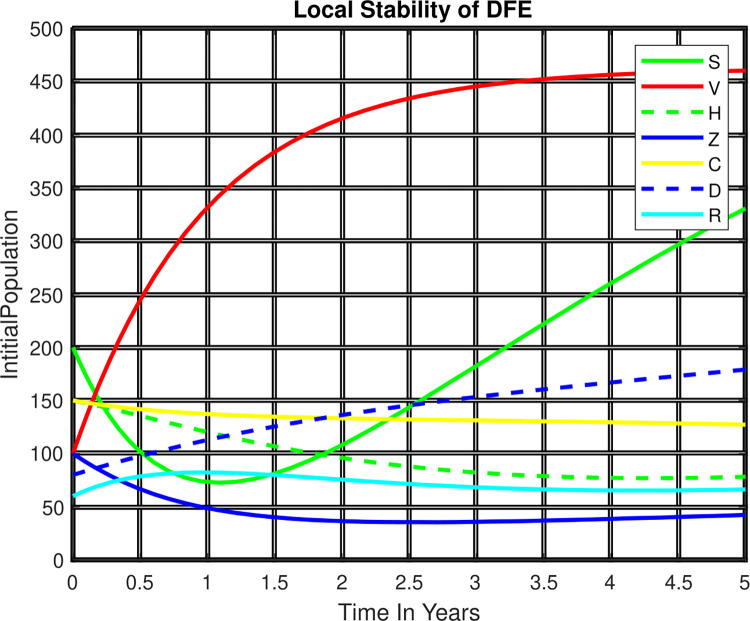
Local stability of EEP.

In Fig 4, we have used numerical simulation for stability analysis of steady state and obtained the result: after a year, the solutions of the HIV and Zoster co-infection dynamical system will tend to the endemic equilibrium point of the model.

**Fig 5:**
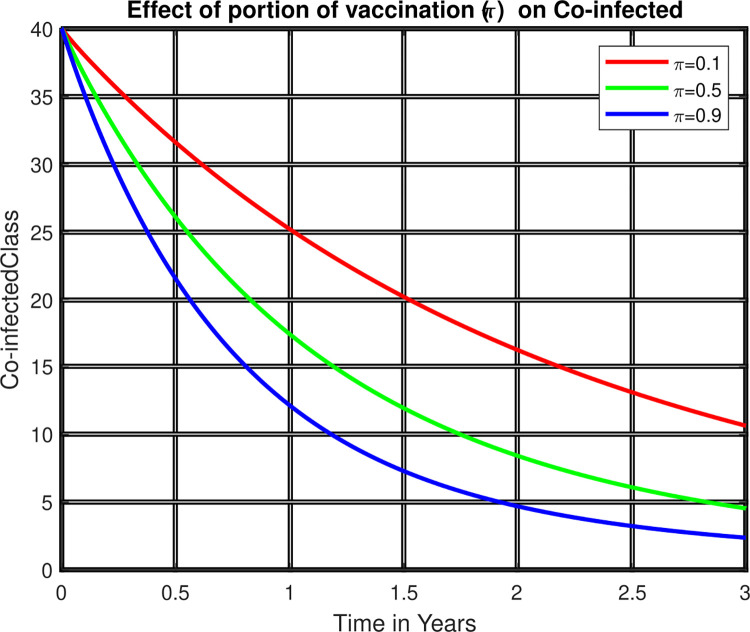
Effect of portion of vaccination co-infected class.

The above Fig 5 shows that as the portion of vaccination for zoster disease increases, the number of individuals affected by both co-infected zoster and HIV declines and so the portion of vaccination can be one of the control measures to be taken to control the transmission of diseases. Moreover, this portion of vaccination can reduce the potential complications arising from the co-infection of HIV and VZV.

**Fig 6:**
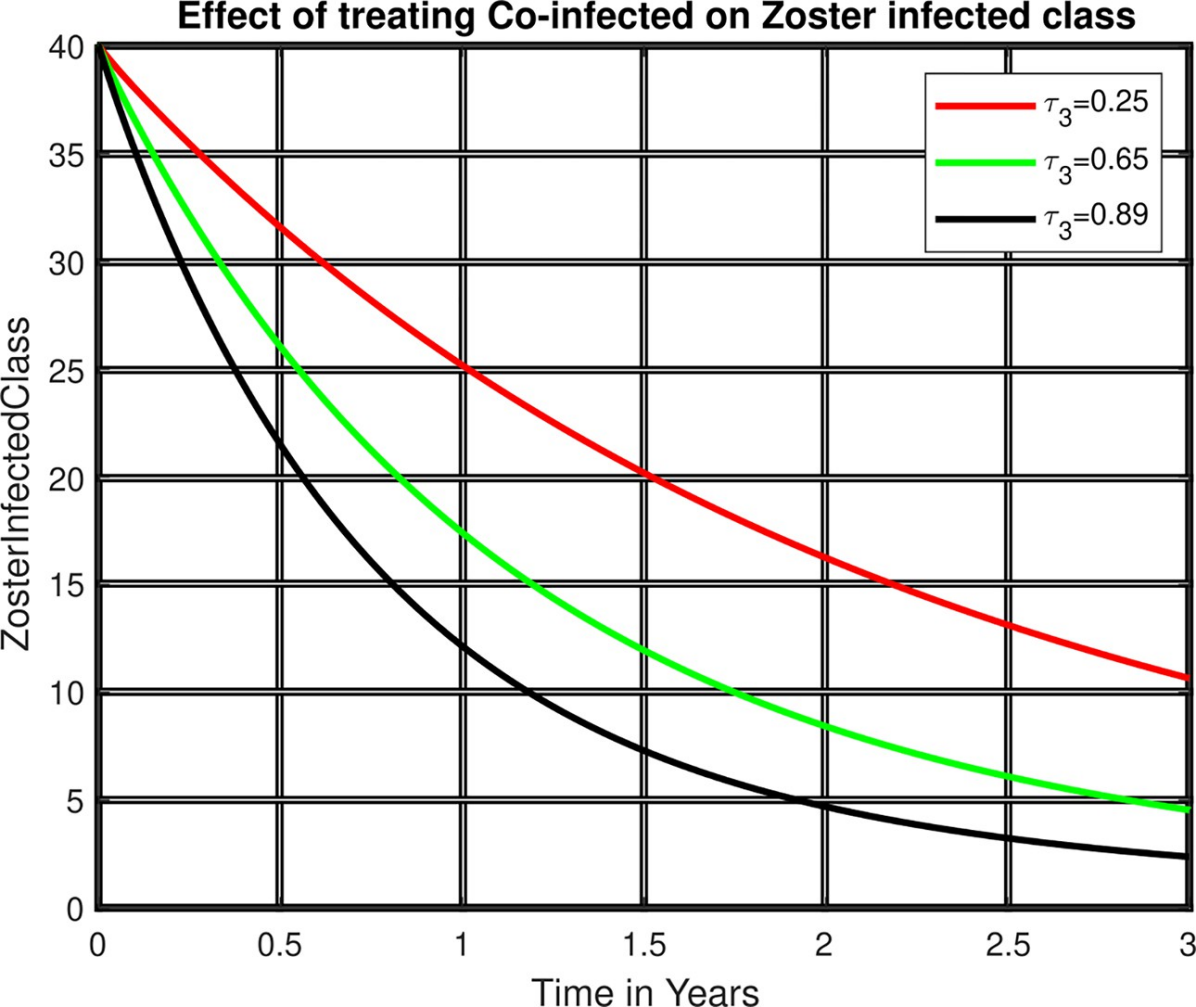
Treatment rate versus zoster infected.

Fig 6 demonstrated that while the treatment for the co-infected class rose, the zoster-infected individuals decreased. As a result, treating co-infection classes is another way of controlling the prevalence of these diseases among the communities.

**Fig 7:**
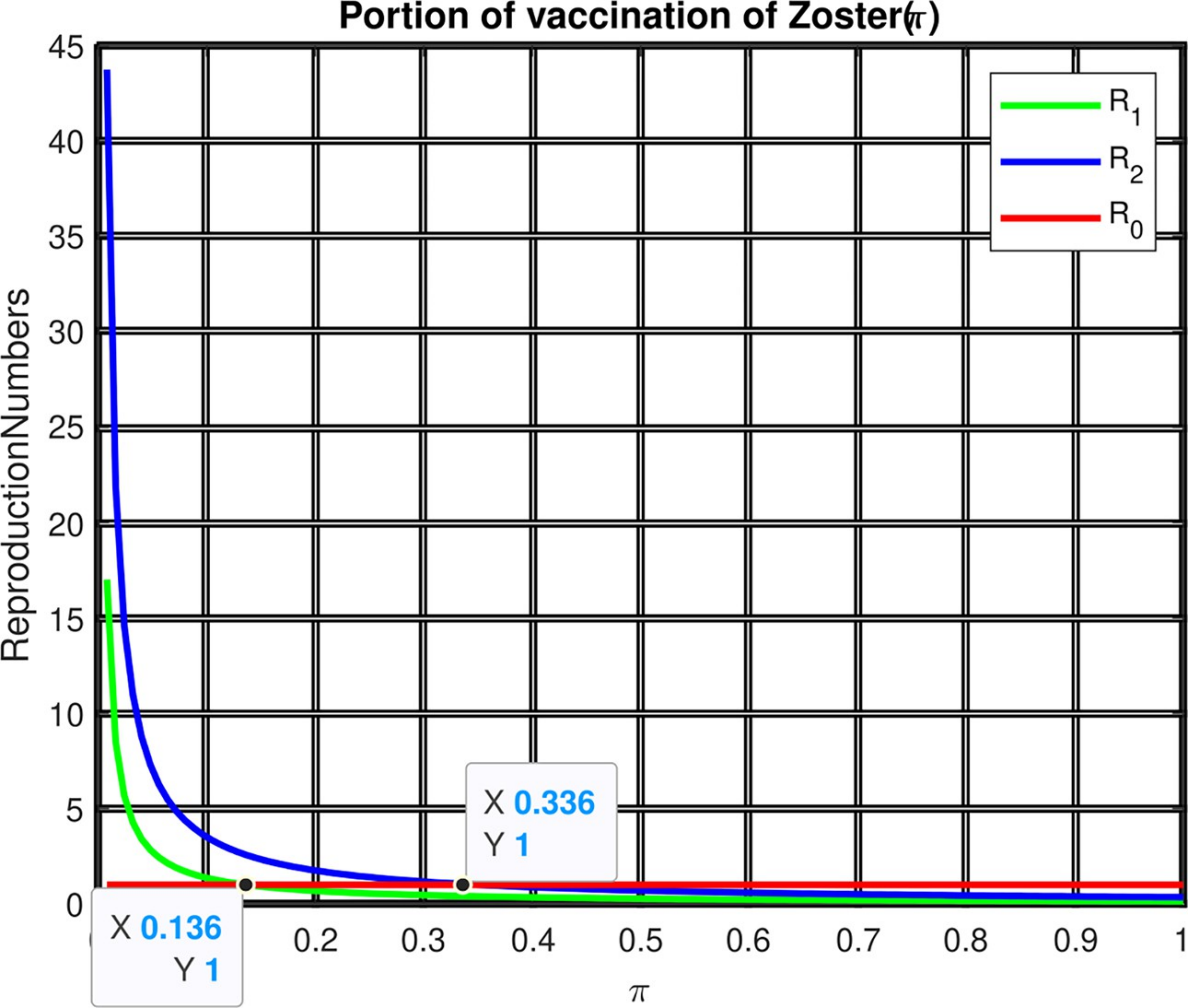
Effect of the portion of vaccination (π) on reproduction numbers.

From Fig 7 we can observe the effect of vaccination (π) on the reproduction number ℛ = *max*{ℛ_1_, ℛ_2_}. The figure reflects that when the value of (π increases, both ℛ_1_ and ℛ_2_ are decreases, which implies that the maximum of them is also declines. Moreover, the value ℛ = *max*{ℛ_1_, ℛ_2_} becomes smaller than one when the value of π>0.336.

**Fig 8:**
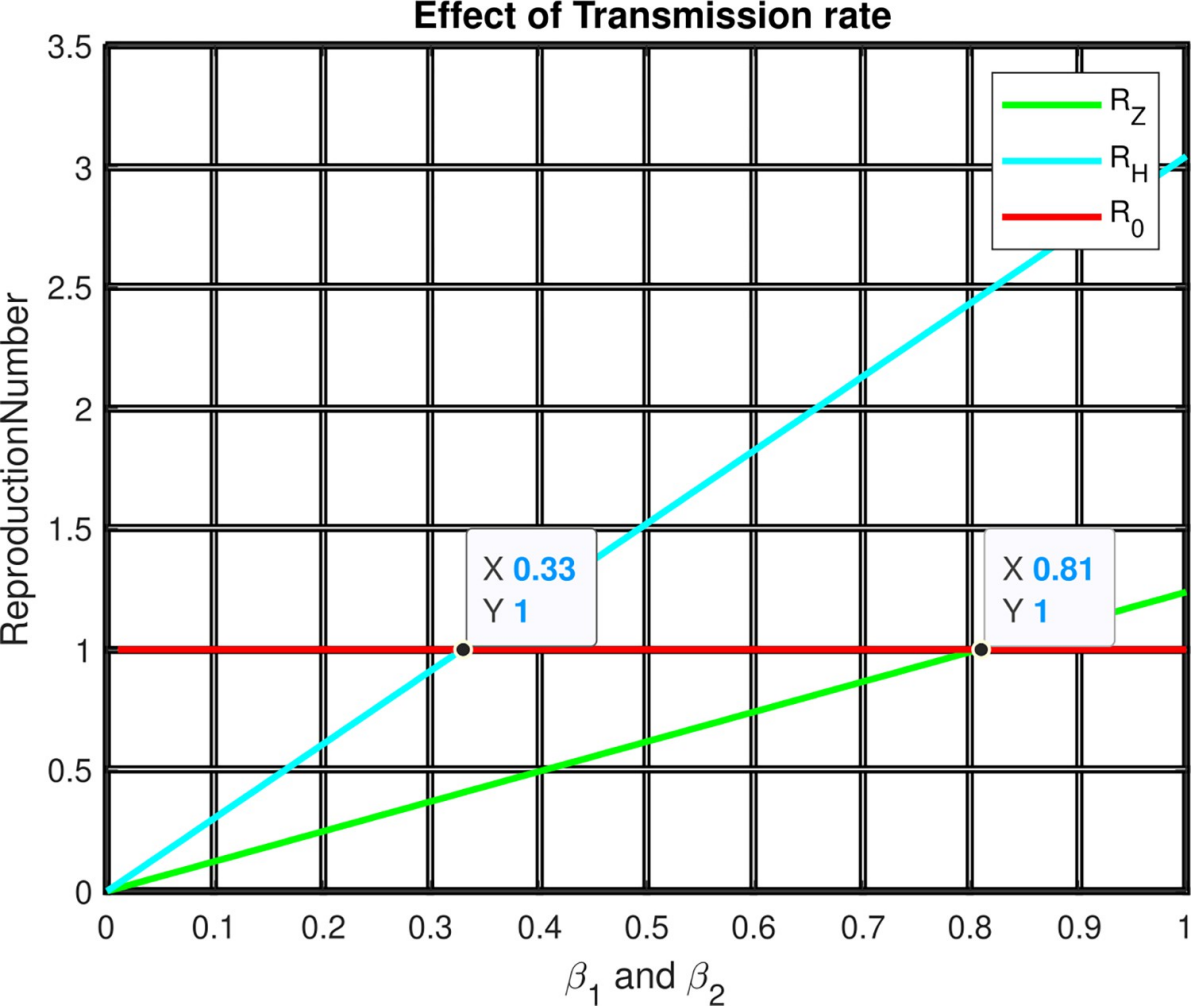
Effect of Transmission rate on reproduction numbers.

Fig 8 1elaborates the investigation of effect of transmission rates {*β*_1_ and *β*_2_} on ℛ = *max*{ℛ_1_, ℛ_2_}. The Figure represents, that as the value of *β*_1_ and *β*_2_ increases, both ℛ_1_ and ℛ_2_ are increases. To have the minimum values of ℛ = *max*{ℛ_1_, ℛ_2_}, the value of ℛ_1_ and ℛ_2_ should be less than 0.33. As a result, we urge all relevant parties to pay close attention to the effective reduction ways for transmission rates for both HIV and Zoster diseases.

**Fig 9:**
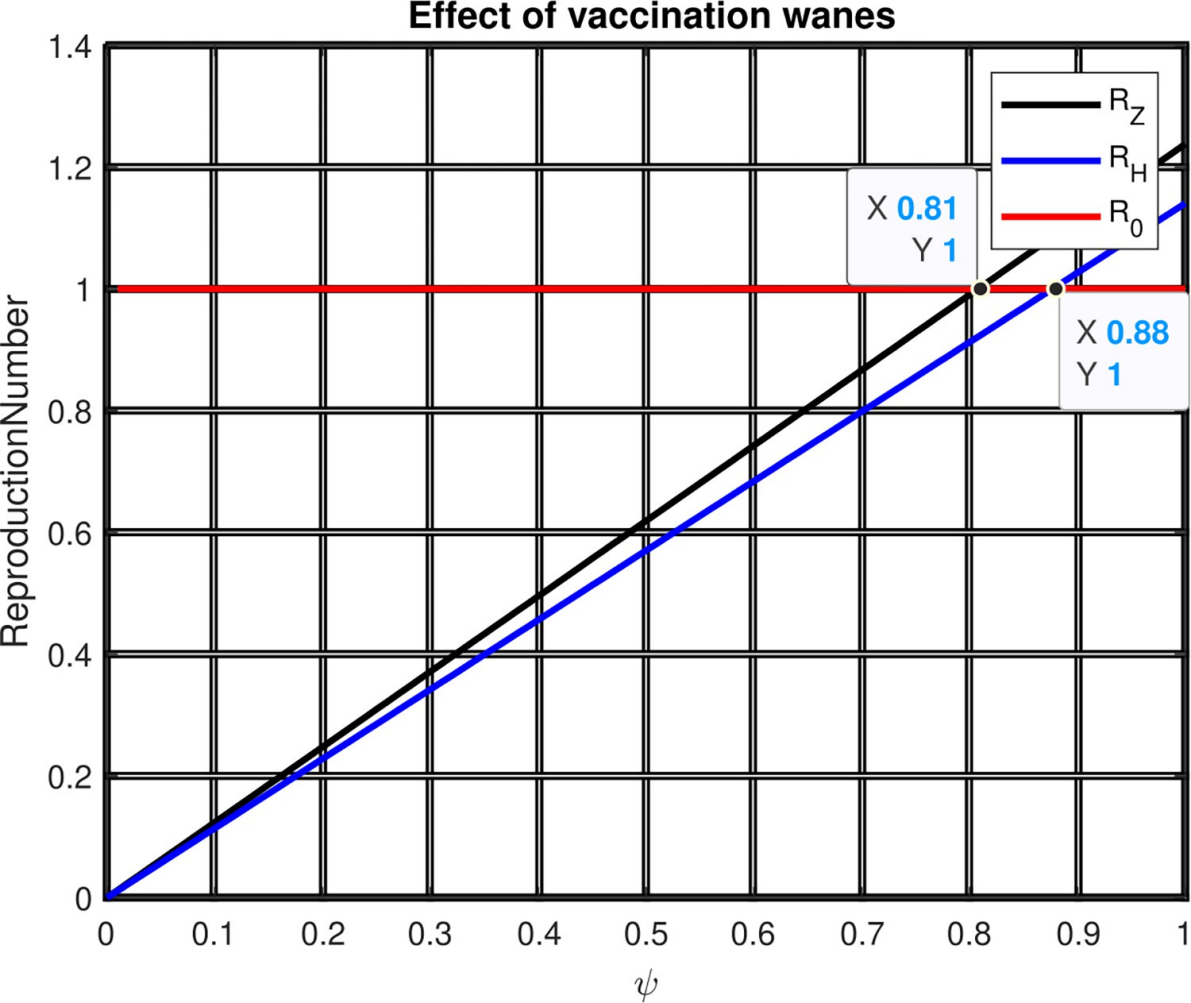
Effect of Zoster vaccination wanes rate on reproduction numbers.

Fig 9 explains the investigation of the effect of Zoster vaccination wanes (*ψ*) on ℛ = *max*{ℛ_1_, ℛ_2_}. The graph illustrates how both ℛ_1_ and ℛ_2_ grow when the value of *ψ* rises. Our conclusion from this finding is that when the value of *ψ* is smaller than 0.81, the minimal value of ℛ = *max*{ℛ_1_, ℛ_2_} = *max*{1.78, 2.76} = 2.76 is reached. The effectiveness of the Zoster vaccination in avoiding the co-infection of zoster and HIV in society should thus be closely monitored, and we encourage all pertinent parties to pay attention to it.

## 4. Result, conclusion, and recommendation

A qualitative evaluation was conducted on the novel deterministic model that was introduced in this work to evaluate the dynamics of HIV co-infection and zoster. The stability of the model’s two nonnegative equilibrium points, DFE and EE, was examined. To ensure that the model had a mathematically and epidemiologically well-posed region, it was examined. Additionally, the reproduction number was ascertained. According to a sensitivity analysis of the model, lowering the positive indices parameters and raising the negative indices parameters was necessary to lower the basic reproduction number and the spread of illness. To investigate how a little parameter change affects reproduction number and state variables, the acquired data were numerically simulated.

The models that have been put forth suggest that one method of reducing HIV and zoster co-infection in the population is medicine. Treating zoster-only cases decreased both infections in the communities, as shown by a computer simulation. Furthermore, expanding access to the Zoster vaccine significantly affects the frequency of both HIV and zoster cases in a particular community because vaccination is a prophylactic measure. Therefore, the following suggestions are hereby given in light of our findings:

- To wipe out the co-infections, an individual has to take a zoster vaccination of more than 33.6%.
- Decreasing the Zoster vaccination wanes by more than 81% can also be one of the controlling measures for the co-existence of these diseases.
- To reduce all three illnesses, vaccine coverage must exceed 33.6%.
- Reducing the spread of co-infection should be managed by decreasing the transmission rate by more than 33%.

The models suggest that administering medicine and treating zoster-only cases can significantly reduce both HIV and zoster co-infections, underscoring the practical impact of pharmaceutical interventions. Moreover, expanding access to the Zoster vaccine emerges as a pivotal prophylactic measure, with computer simulations illustrating its tangible effect on diminishing the frequency of both diseases within a community. These outcomes not only strengthen the case for vaccination programs but also emphasize the real-world implications of public health measures.

For future research, it is advisable to consider additional interventions like targeted antiretroviral therapy for HIV, explore the impact of behavioral changes through education, and investigate the influence of spatial factors on co-infection dynamics. Converting the model into a fractional order could enhance its accuracy, capturing more complex real-world scenarios like [14,21]. Further analysis of optimal vaccination strategies, including timing and frequency, would be beneficial, along with evaluating the economic and social implications of co-infections. Additionally, researchers may explore long-term immunity dynamics following vaccination or infection to understand co-infection persistence. These recommendations aim to enrich our understanding of HIV and zoster co-infections, offering insights for more effective public health interventions in the future.

## Supporting information

S1 File (DOCX)
