# Supplementary material for: A mathematical model for varicella-zoster and HIV co-dynamic supported by numerical simulations
Source: PLoS One. 2024 Mar 1;19(3):e0299734. doi: 10.1371/journal.pone.0299734 (PMC10906872; doi:10.1371/journal.pone.0299734)
Supplement: S1 File — (DOCX) [file pone.0299734.s001.docx]

The values of each parameters in the Table below has been collected from published paper cited and referenced below.

| Parameters | Description | values | Unit | Sources |
| --- | --- | --- | --- | --- |
| $\mu$ | Human natural mortality rate | 0.1 | $\mathrm{Time}^{-1}$ | [14] |
| $\bigwedge$ | Human recruitment rate | 100 | ${Size*Time}^{-1}$ | [16,17] |
| $\pi$ | Portion of vaccination of Zoster | 0.8 | $\mathrm{Time}^{-1}$ | Estimated from [18,19] |
| $\psi$ | Vaccination wanes rate | 0.35 | $\mathrm{Time}^{-1}$ | Estimated |
| $a$ | Modification | 1 | $\mathrm{Time}^{-1}$ | Estimated |
| $b$ | Modification | 1 | $\mathrm{Time}^{-1}$ | Estimated |
| $\delta_{1}$ | zoster death rate | 0.32 | $\mathrm{Time}^{-1}$ | Estimated from [17] |
| $\delta_{2}$ | HIV death rate for Unaware |  | $\mathrm{Time}^{-1}$ |  |
| $\delta_{3}$ | zoster and HIV co-infection death rate | 0.3 | $\mathrm{Time}^{-1}$ | Estimated from [14, 17] |
| $\gamma$ | The rate at which Zoster recovered individual re-infected | 0.65 | $\mathrm{Time}^{-1}$ | Estimated from [17] |
| $\rho$ | The rate at which HIV infected with undetectable viral load become re-infected by zoster | 0.32 | $\mathrm{Time}^{-1}$ | Estimated |
| $\beta_{1}$ | HIV Transmission rate | 2.5 | ${Size*Time}^{-1}$ | Estimated from [18] |
| $\beta_{2}$ | Zoster Transmission rate | 3.5 | ${Size*Time}^{-1}$ | [17] |
| $\tau_{1}$ | Zoster treatment rate | 0.87 | $\mathrm{Time}^{-1}$ | Estimated from [15] |
| $\tau_{2}$ | HIV treatment rate | 0.45 | $\mathrm{Time}^{-1}$ | Estimated from [20] |
| $\tau_{3}$ | The rate at which Zoster and HIV co-infected individuals treated | 0.53 | $\mathrm{Time}^{-1}$ | Estimated from [19] |
| 𝜎 | Probability of death of newborns infected with HIV at birth | 0.35 | $\mathrm{Time}^{-1}$ | Estimated |
| 𝜑 | Rate of Mother to Child Transmission rate of HIV | 0.6 | $\mathrm{Time}^{-1}$ | Estimated |

References

1. *Wodajo, Firaol Asfaw, and Temesgen Tibebu Mekonnen. "Effect of Intervention of Vaccination and Treatment on the Transmission Dynamics of HBV Disease: A Mathematical Model Analysis." Journal of Mathematics 2022 (2022).*
2. *Teklu, Shewafera Wondimagegnhu, and Belela Samuel Kotola. "A dynamical analysis and numerical simulation of COVID-19 and HIV/AIDS co-infection with intervention strategies." Journal of Biological Dynamics 17, no. 1 (2023): 2175920.*
3. *Karsai, János, Rita Csuma-Kovács, Ágnes Dánielisz, Zsuzsanna Molnár, János Dudás, Teodóra Borsos, and Gergely Röst. "Modeling the transmission dynamics of varicella in Hungary." Journal of Mathematics in Industry 10, no. 1 (2020): 12.*
4. *Karsai, János, Rita Csuma-Kovács, Ágnes Dánielisz, Zsuzsanna Molnár, János Dudás, Teodóra Borsos, and Gergely Röst. "Modeling the transmission dynamics of varicella in Hungary." Journal of Mathematics in Industry 10, no. 1 (2020): 12.*
5. *Kennedy, Peter GE, Trine H. Mogensen, and Randall J. Cohrs. "Recent issues in varicella-zoster virus latency." Viruses 13, no. 10 (2021): 2018.*
6. *Patil, Anant, Mohamad Goldust, and Uwe Wollina. "Herpes zoster: a review of clinical manifestations and management." Viruses 14, no. 2 (2022): 192.*
7. *Teklu, Shewafera Wondimagegnhu. "Investigating the Effects of Intervention Strategies on Pneumonia and HIV/AIDS Coinfection Model." BioMed Research International 2023 (2023).*
8. *Kotola, Belela Samuel, Dawit Melese Gebru, and Haileyesus Tessema Alemneh. "Appraisal and Simulation on Codynamics of Pneumonia and Meningitis with Vaccination Intervention: From a Mathematical Model Perspective." Computational and Mathematical Methods in Medicine 2022 (2022).*
9. *Teklu, Shewafera Wondimagegnhu, and Belela Samuel Kotola. "The Impact of Protection Measures and Treatment on Pneumonia Infection: A Mathematical Model Analysis Supported by Numerical Simulation." bioRxiv (2022): 2022-02.*
10. *Teklu, Shewafera Wondimagegnhu, and Koya Purnachandra Rao. "HIV/AIDS-Pneumonia Codynamics Model Analysis with Vaccination and Treatment." Computational and Mathematical Methods in Medicine 2022 (2022).*
11. *Maayah, Banan, Omar Abu Arqub, Salam Alnabulsi, and Hamed Alsulami. "Numerical solutions and geometric attractors of a fractional model of the cancer-immune based on the Atangana-Baleanu-Caputo derivative and the reproducing kernel scheme." Chinese Journal of Physics 80 (2022): 463-483.*
12. *Kennedy, Peter GE, and Trine H. Mogensen. "Determinants of neurological syndromes caused by varicella zoster virus (VZV)." Journal of NeuroVirology 26 (2020): 482-495.*
13. *Yusuf, Abdullahi, Sania Qureshi, Umar T. Mustapha, Salihu S. Musa, and Tukur A. Sulaiman. "Fractional modeling for improving scholastic performance of students with optimal control." International Journal of Applied and Computational Mathematics 8, no. 1 (2022): 37.*
14. *Teklu, Shewafera Wondimagegnhu, and Belela Samuel Kotola. "Insight into the Treatment Strategy on Pneumonia Transmission with Asymptotic Carrier Stage using Fractional Order Modelling Approach." Computer Methods and Programs in Biomedicine Update (2024): 100134*
15. *Kennedy, Peter GE. "The Spectrum of Neurological Manifestations of Varicella–Zoster Virus Reactivation." Viruses 15, no. 8 (2023): 1663.*
16. *Marais, Gert, Michelle Naidoo, Kate McMullen, Alan Stanley, Alan Bryer, Diederick van der Westhuizen, Kathleen Bateman, and Diana Ruth Hardie. "Varicella‐zoster virus reactivation is frequently detected in HIV‐infected individuals presenting with stroke." Journal of medical virology 94, no. 6 (2022): 2675-2683.*
17. *Baird, Nicholas L., Shuyong Zhu, Catherine M. Pearce, and Abel Viejo-Borbolla. "Current in vitro models to study varicella zoster virus latency and reactivation." Viruses 11, no. 2 (2019): 103.*
18. *Chen, J., Li, F., Tian, J., Xie, X., Tang, Q., Chen, Y. and Ge, Y., 2023. Varicella zoster virus reactivation following COVID‐19 vaccination in patients with autoimmune inflammatory rheumatic diseases: A cross‐sectional Chinese study of 318 cases. Journal of Medical Virology, 95(1), p.e28307.*
19. *Tommasi, Cristina, and Judith Breuer. "The biology of varicella-zoster virus replication in the skin." Viruses 14, no. 5 (2022): 982.*
20. *Hertel, M., M. Heiland, S. Nahles, M. Von Laffert, C. Mura, P. E. Bourne, R. Preissner, and S. Preissner. "Real‐world evidence from over one million COVID‐19 vaccinations is consistent with reactivation of the varicella‐zoster virus." Journal of the European Academy of Dermatology and Venereology 36, no. 8 (2022): 1342-1348.*
